# Mucus organisation is shaped by colonic content; a new view

**DOI:** 10.1038/s41598-017-08938-3

**Published:** 2017-08-17

**Authors:** J. B. J. Kamphuis, M. Mercier-Bonin, H. Eutamène, V. Theodorou

**Affiliations:** Neuro-Gastroenterology and Nutrition Team, UMR 1331, INRA Toxalim, INP-EI-Purpan, Université de Toulouse, Toulouse, France

## Abstract

The colonic mucus barrier is commonly described as a continuous double layer covering the epithelium, separating the microbiota from the intestinal tissue. This model is currently considered valid throughout the colon. The colon is characterised by regional anatomo-functional specificities such as presence and consistency of contents and location. In this study, we characterised the organisation of the colonic mucus barrier in proximal and distal colon of rodents by histological and FISH staining, taking into account aforementioned specificities. By using longitudinal sections and imaging extensive areas of tissue with and without colonic contents, we have obtained a spatiotemporal overview of mucus organisation in the colon. We describe for the first time that the colonic mucus layer covers the faeces instead of the epithelium in the distal colon. This faecal mucus layer confines the microbiota to the faeces and prevents it from remaining in empty distal colon. In the proximal colon, the mucus did not form a separating layer between bacteria and epithelium. We conclude that the organisation of colonic mucus is reliant on the presence of the colonic content, and the location within the colon. Our findings reopen the discussion on the nature of the colonic mucus barrier.

## Introduction

The intestinal mucus layer has a critical role in gut health. It facilitates the passage of faeces through the intestine, reducing the risk of damage to the gut epithelium^1^. The critical importance of intestinal mucus to gut health is underlined by the spontaneous development of colitis in Muc2 knockout (Muc2^−/−^) mice^2^, and their increased susceptibility to pathogens^3^. Bacteria of the intestinal microbiota profit the host with their metabolic activities^4^, and modulation of the immune system^5^. Limiting the contact between intestinal microbes and the colonic epithelium, a thick mucus layer is described that separates the two^6^. Johansson *et al*.^7^ reported that the distal colonic mucus layer is organised in 2 parts, a firm component and a loose one, both built around Muc2 mucin protein. This layer has been described to be organised as a loose layer inhabited by bacteria, and a layer firmly attached to the epithelium, devoid of bacteria^7^. Primarily, this understanding is based on histological observations of transversal sections of colon, imaging the mucus layers separating microbes from the epithelium. It became evident that it was impossible to image the mucus barrier in histological sections lacking a faecal pellet, so the method of reference became to section samples containing a faecal pellet to investigate the properties of the mucus layer. A given explanation for this necessity was that the faecal pellet protects and conserves the mucus barrier during histological processing^7^. Additionally, to further investigate mucus barrier properties, *ex vivo* experiments involving explant tissue^8^, and *in vivo* experiments have been designed and performed^6, 9^. The general organisation is described to be constantly sustained^7^, though specifics are variable over time, and influenced by harmful factors such as inflammation^10^ or even the time frame of microbiota colonisation^11^, emphasising the intimate relationship between intestinal mucus and gut microbiota. Furthermore, microscopic analysis of the biostructure of human faeces shows mucus irregularly intersecting into the faecal mass^12^, and a mucus layer is observed on expelled faeces from rodents as well as humans^12, 13^. These results obtained from faecal material illustrate the organisation of the contents of distal colon, which does not necessarily reflect the situation in the proximal colon. Apart from the morphological differences between the proximal and distal colon, the contents too are dissimilar in their consistency and humidity; the proximal colon content is liquid whereas the contents of distal colon normally form pellets with a firmer consistency. Changes in the physical and biochemical properties of colonic mucus have been documented in pathophysiological conditions. For instance, colonic mucus from ulcerative colitis (UC) patients in the active phase of the disease is thinner and more penetrable to fluorescent beads compared to healthy subjects^14^ and glycosylation of colonic mucins is found to be correlated with the severity of inflammation in UC patients^15^. Likewise, O-glycosylation of mucins is strongly affected by chronic psychological stress in rats, associated with a flattening and a loss of cohesive properties of the mucus layer^16^. Despite data indicating that the properties of intestinal mucus are dynamic and influenced by a disruption of intestinal homeostasis, modification of the microbiota, altered food intake, and disturbances in gut motility, little is known about the dynamics of the mucus barrier during normal gastrointestinal transit, reflected by the periodic presence and absence of faeces. This study is meant to clarify the influence of the colonic load on mucus layer structure and organisation in physiological conditions in the colon. We performed longitudinal and transversal sections of distal and proximal colon of rat and mouse in samples covering regions with and without luminal content. We used classical histology to characterise tissue and mucus morphology, combined with ‘Fluorescent *in situ* Hybridisation’ (FISH) staining to localise the gut microbiota. Instead of depicting select microscopic fields, we generated images covering the entirety of both transversal and longitudinal sections using a Manual Ultra-high resolution Composite Image Overview (MUCIO) approach, creating comprehensive overviews of tissue sections, rendering them more understandable as well as preventing an image selection bias in our results.

## Results

### Organisation of mucus in distal colon is influenced by the presence of colonic content

**Figure 1:**
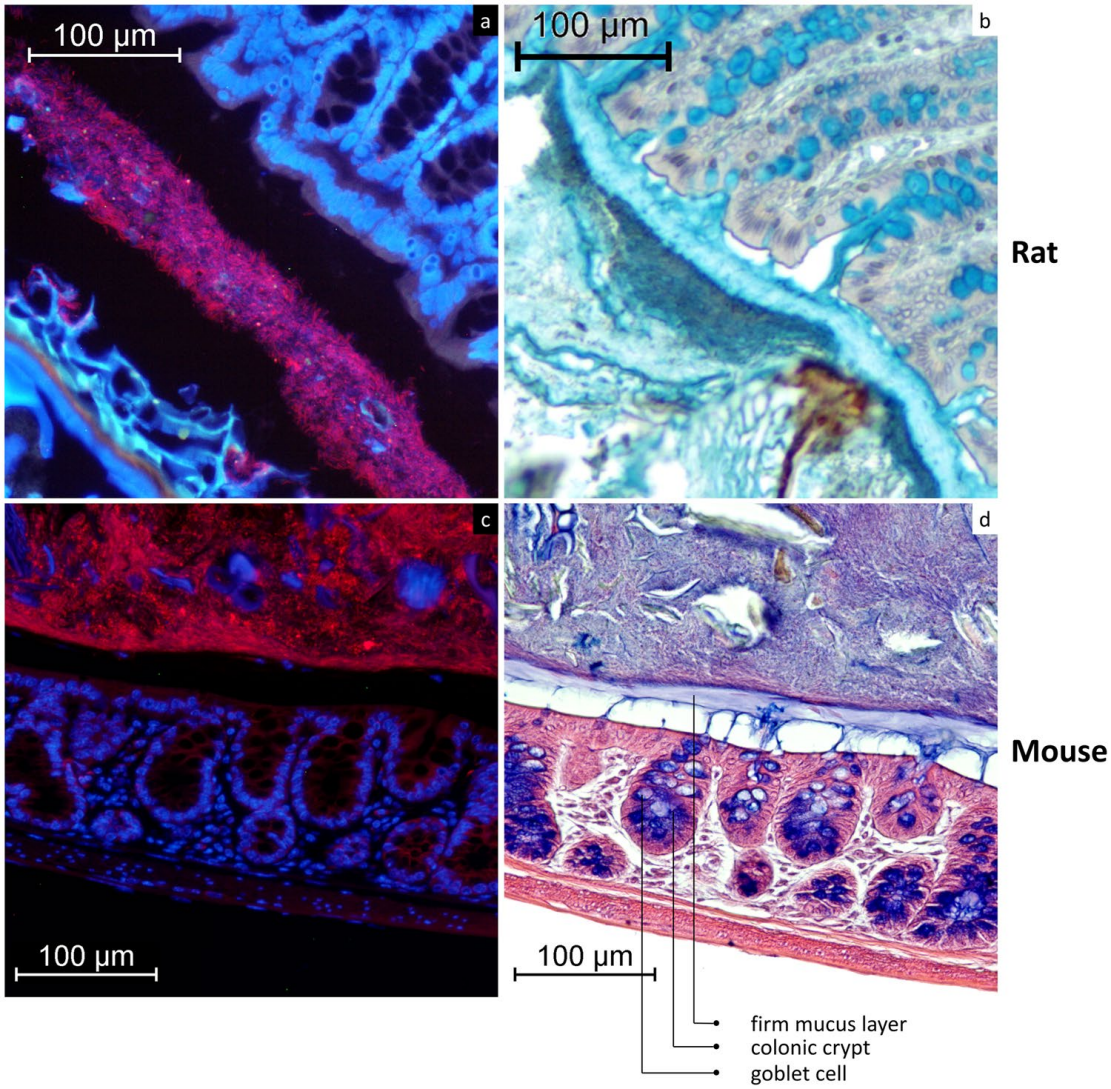
A firm mucus layer separates the bacteria from the epithelium. (**a**) FISH stained transversal section (Bacteria: red; nuclear staining DAPI: blue) of rat distal colon. (**b**) AB/H/E stained transversal section of rat distal colon. (**c**) FISH stained transversal section (Bacteria: red; nuclear staining DAPI: blue) of mouse distal colon. (**d**) AB/H/E stained longitudinal section of mouse distal colon. The FISH staining indicates where the bacteria are located, and that this corresponds to the dark blue/purple staining in AB/H/E stained samples.

**Figure 2:**
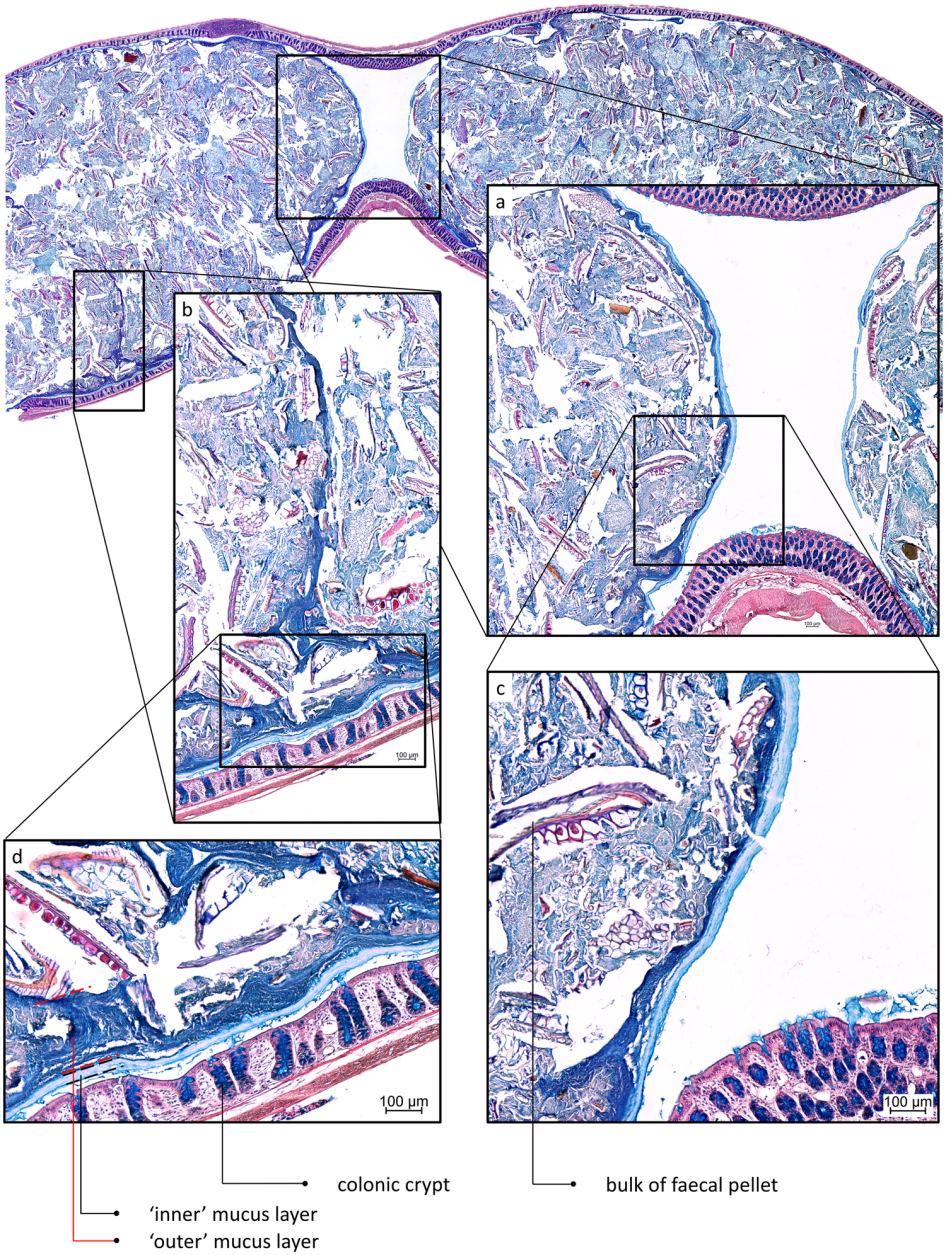
AB/H/E stained longitudinal section of distal rat colon containing faecal pellets. A sterile mucus layer covers the pellet completely. (**a**) The mucus layers attached to the faeces cover the entire pellet. (**b**) A line of bacteria-overgrown mucus penetrates the faecal pellet. (**c**) Sterile (light blue) and bacteria-overgrown mucus layers (dark blue) can be recognised, mucus production by goblet cells in the lumen is visible. (**d**) Sterile (‘inner’) and bacteria-overgrown (‘outer’) mucus layers, are recognised in a manner commonly observed in transversal sections.

**Figure 3:**
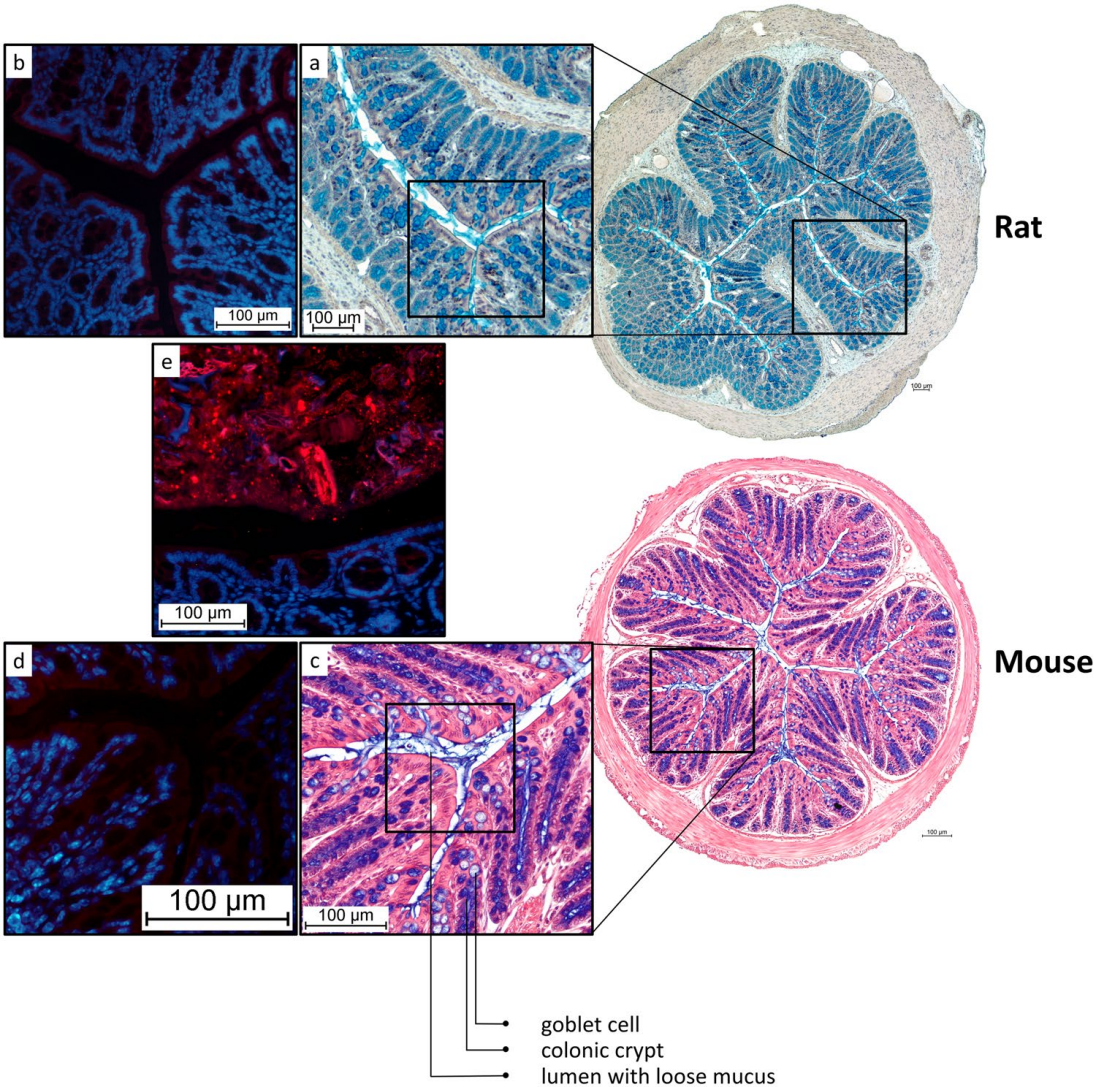
AB/H/E stained transversal sections of distal rat and mouse colon without faeces. The lumen contains loose mucus, but no bacteria are detected. (**a**) Close-up of rat empty distal colon (**b**) FISH staining indicating the absence of bacteria in the lumen of collapsed rat colon. (**c**) Close-up of mouse empty distal colon. (**d**) FISH staining indicating the absence of bacteria in the lumen of collapsed mouse colon. (**e**) FISH staining of rat colon containing faeces (Bacteria: red; nuclear staining DAPI: blue) serving as a positive control.

**Figure 4:**
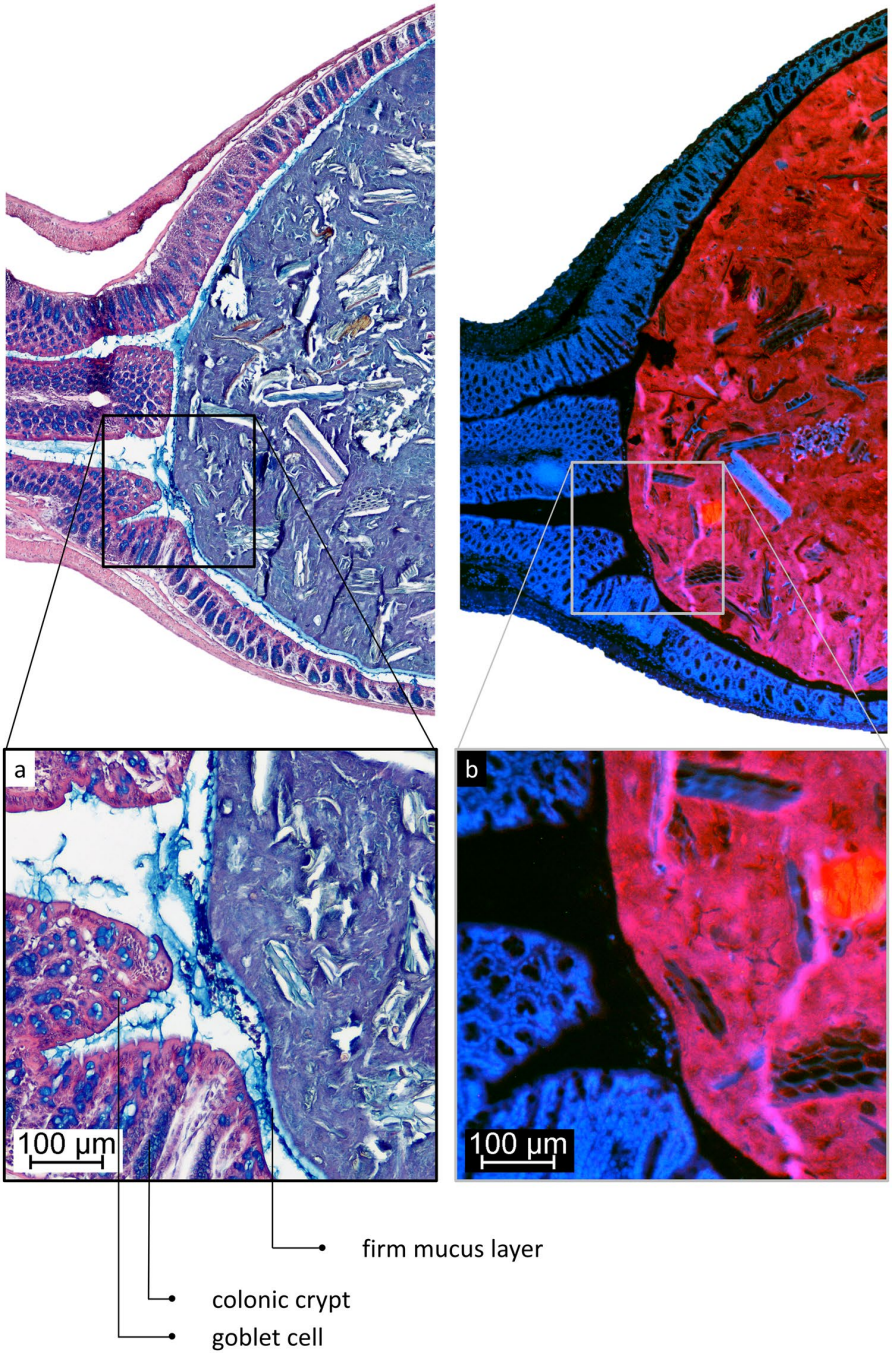
In distal colon, the microbiota is confined to the faecal pellet. (**a**) AB/H/E stained longitudinal section of mouse distal colon. (**b**) FISH stained (Bacteria in red; nuclei in blue) longitudinal section of mouse distal colon. The bacteria are located in the faecal pellet, the lumen of the empty colonic part is almost completely devoid of bacteria, but does contain some loose mucus. A mucus layer devoid of bacteria, confining the bacteria to the faeces, covers the pellet.

**Figure 5:**
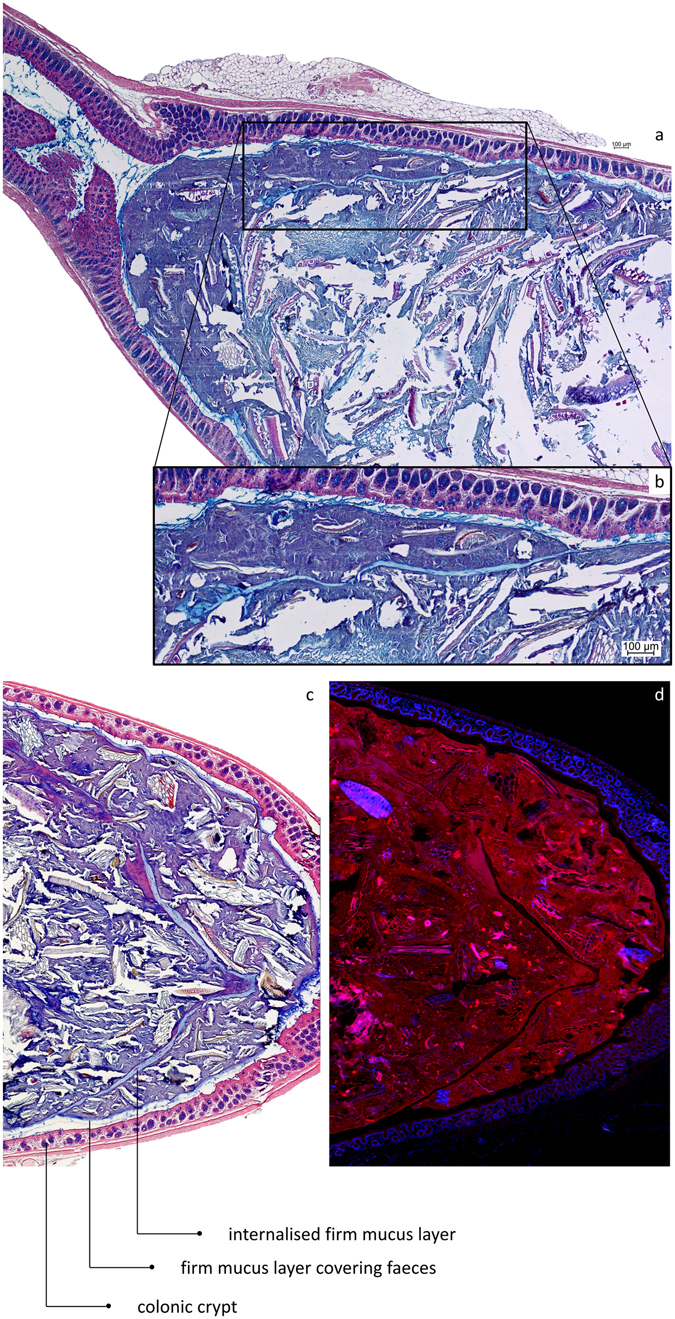
After collisions between faecal pellets in the distal colon, the mucus layers covering their surface can be internalised into the resulting composite pellet. (**a**) AB/H/E stained longitudinal section of distal mouse colon. (**b**) Close-up showing the internalised firm mucus layer. (**c**) AB/H/E stained longitudinal section of distal mouse colon. A firm mucus layer devoid of bacteria is found inside the pellet. (**d**) FISH stained (Bacteria in red; nuclei in blue) longitudinal section of mouse distal colon, the internalised firm mucus layer is recognised by the absence of bacteria.

In transversal sections of full distal colon, a sterile mucus barrier separated the epithelium from the faeces and the microbiota (Fig. 1). Bacteria were almost absent from this layer of about 35 µm (Fig. 1a,b) in rat, and 20 µm (Fig. 1c,d) in mouse. Changing the direction of sectioning, in longitudinal sections covering both empty and full sections of rat distal colon, faecal pellets were completely covered by a sterile mucus layer of variable thickness (36 µm ± 9 µm), with a mix of bacteria and mucus present underneath (Fig. 2a–d). The mucus layer only covers the faecal pellet, and is not attached to the epithelium (Fig. 2a,c). The dark blue layer (Fig. 2c,d) is rich in bacteria, as corroborated by Fig. 1. In transversal sections of empty distal colon mucus was not organised in a similar fashion; instead, ‘luminal’ mucus without an apparent layer structure was present (Fig. 3). In these empty sections the tissue was folded up neatly, with opposing epithelia very close to each other, sometimes in contact. In rat tissue, the distance between opposite epithelia in empty distal colon averages 17 ± 9 µm (Fig. 3a), in mice it averages 16 ± 7 µm (Fig. 3c). In contrast to the situation in colon containing faeces, we have not observed bacteria-colonised mucus in empty distal colon (Fig. 3b,d). A longitudinal section of mouse distal colon containing faeces shows that bacteria are confined to the pellet, which consists mostly of alimentary residues embedded in microbiota-colonised material (Fig. 4). The sterile mucus layer can be internalised by the colonic contents in mouse distal colon (Fig. 5). In this situation, the internalised firm mucus layer remains sterile, seen from the lack of signal in a FISH staining (Fig. 5c,d).

### Excreted faeces are covered by a faecal mucus layer

**Figure 6:**
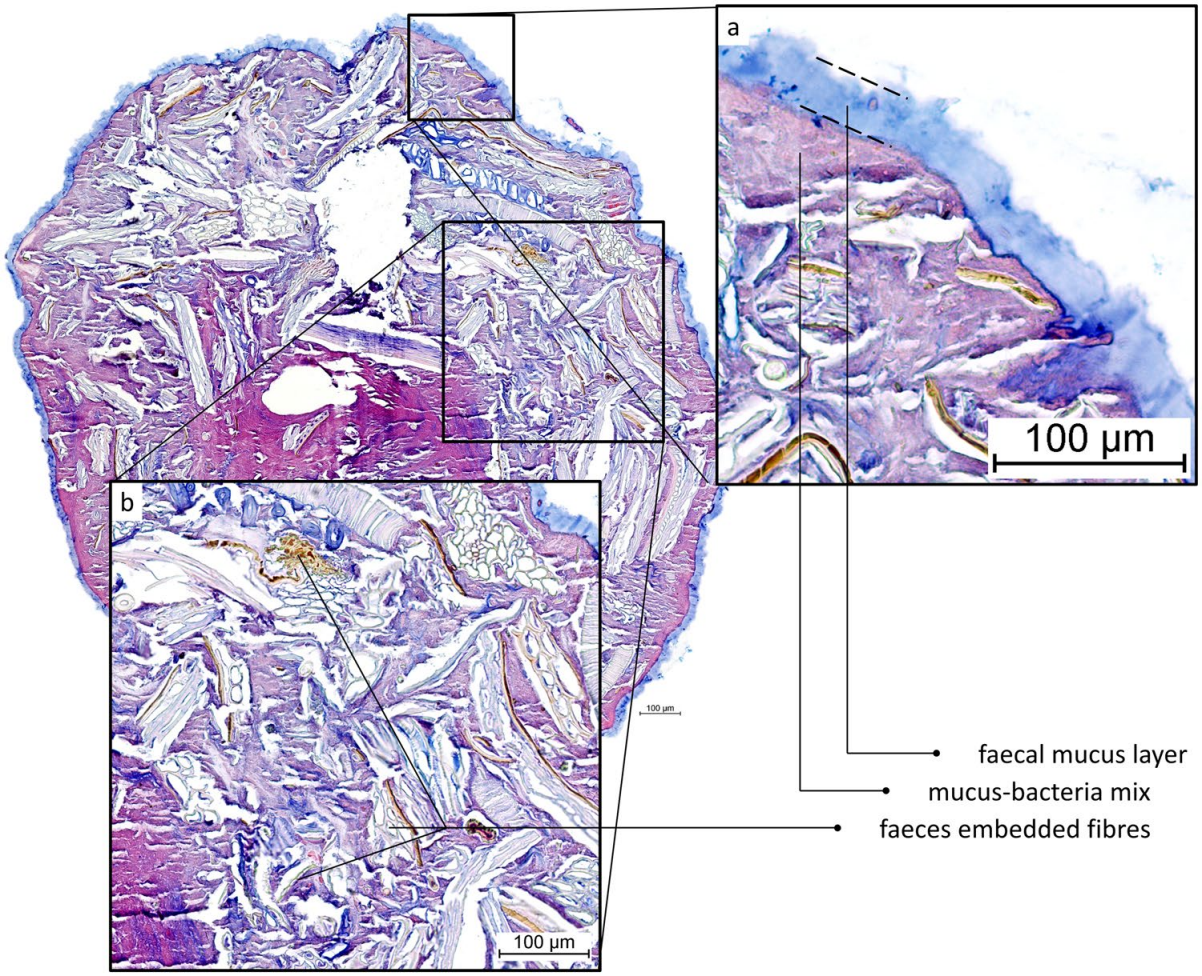
AB/H/E stained transversal cross-section of an expelled mouse faecal pellet. (**a**) A mucus layer devoid of bacteria covers the entire pellet. (**b**) Apart from alimentary residues and microbiota mixed with mucus, there are undigested plant fibres embedded in the faeces, as commonly observed in faecal matter in the gut as well.

On expelled faecal pellets of mice, a mucus layer (24 ± 4.5 µm) devoid of bacteria covers a mix of bacteria and mucus directly (Fig. 6), as well as the rest of the faecal material. This faecal mucus layer has the same appearance and general thickness as the one observed previously (Figs 1 and 2) in distal colon, which covers faecal pellets and separates contents from the epithelium.

### Mucus barrier in proximal colon is affected by consistency of contents

**Figure 7:**
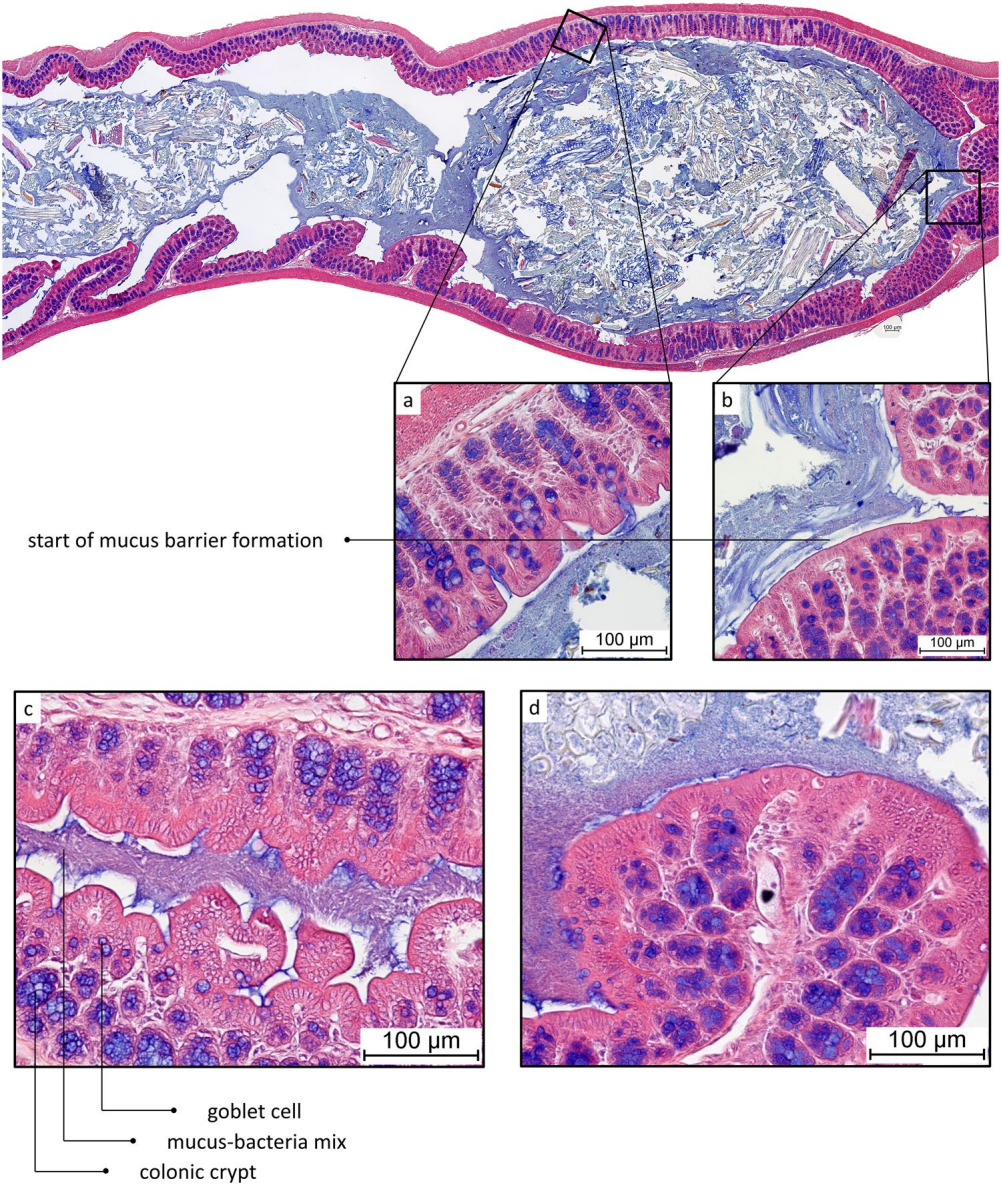
AB/H/E stained longitudinal section of mouse proximal colon. Proximal side of tissue on the left, distal on the right. (**a**) Close-up of proximal part of newformed pellet. (**b**) Close-up of distal part of newformed pellet. (**c**) AB/H/E stained longitudinal section of mouse proximal colon. No mucus layer separates bacteria from epithelium. (**d**) AB/H/E stained longitudinal section of mouse proximal colon. No mucus layer separates bacteria from epithelium.

**Figure 8:**
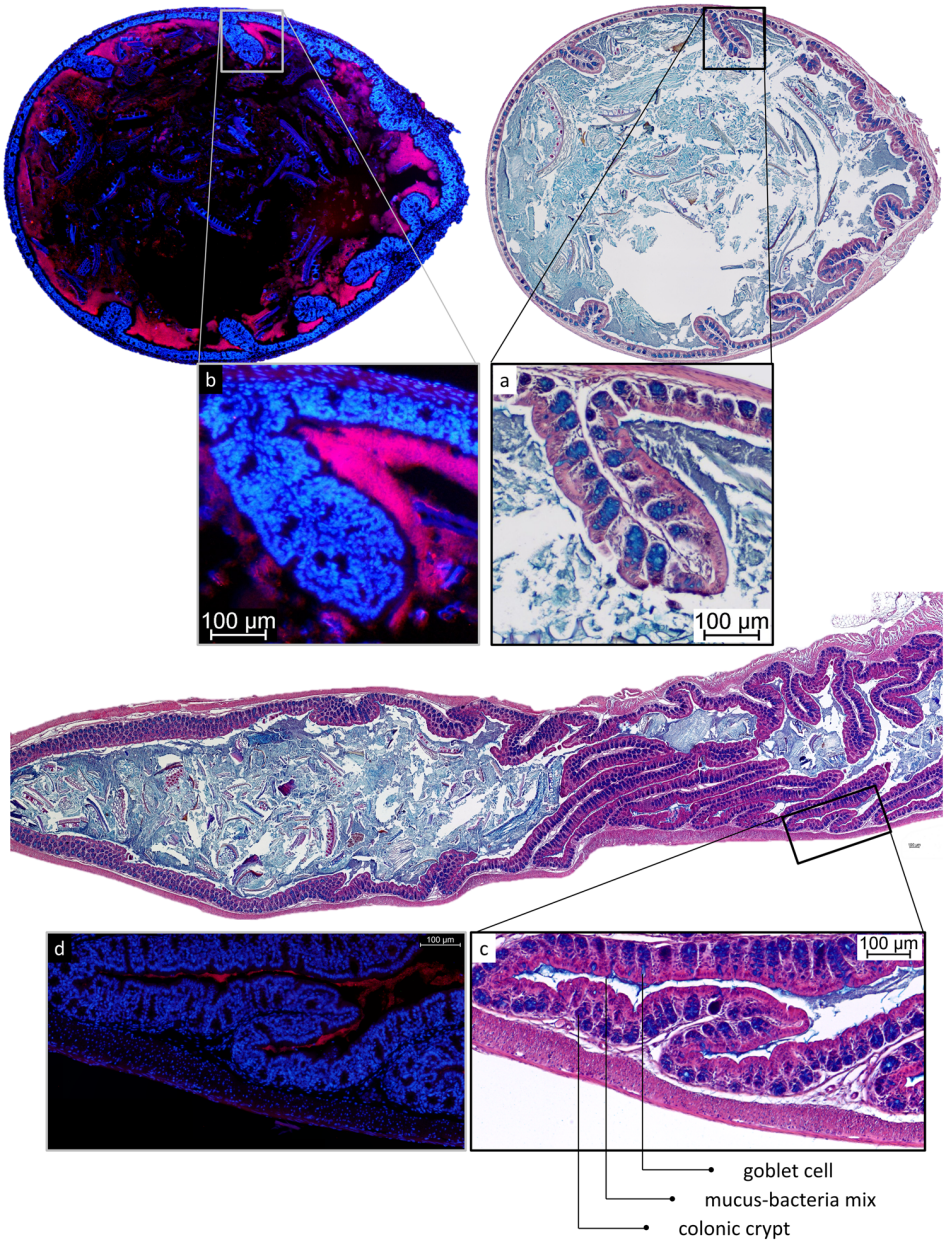
Localisation of microbiota in mouse proximal colon. (**a**) AB/H/E staining of transversal section of mouse proximal colon. (**b**) FISH staining of consecutive transversal section of mouse proximal colon. Bacteria are observed in direct contact with the epithelium (**a**), without a mucus layer to separate them from the tissue (**a**,**b**). Significantly less bacteria are observed in the middle of the contents than near to the mucosa. A high concentration of bacteria is detected particularly under the tissue folds specific to the proximal colon. (**c**) AB/H/E staining of longitudinal section of mouse proximal colon. (**d**) FISH staining of consecutive longitudinal section of mouse proximal colon. In collapsed proximal colon, bacteria mixed with mucus remain present in the lumen of folded tissue.

In the proximal colon, the microbiota was in contact with the epithelium with a noticeable absence of a significant mucus barrier (Fig. 7). The colonic contents were seen to form the first pellet in the distal end of the proximal colon, on the transition towards the distal colon. Here, the first establishment of a mucus barrier can be observed, in multiple streaks on the most distal part of the newly formed pellet (Fig. 7b). In proximal colon, mucus containing bacteria was found in direct contact with the colonic tissue, both in more folded collapsed tissue (Fig. 7c) and in tissues where the lumen is distended by colonic contents (Fig. 7d). In mostly or completely collapsed proximal colon, bacteria-colonised mucus was found in contact with the intestinal tissue, unseparated by a sterile mucus barrier (Figs 7c and 8c,d), in contrast to the situation described earlier for distal colon. In full proximal colon, we observed bacteria in contact with the epithelium too, with the microbiota present in an increasing gradient towards the tissue (Figs 7d and 8a,b); the highest concentration of bacteria was present around and especially below the tissue folds specific to the proximal colon.

## Discussion

**Figure 9:**
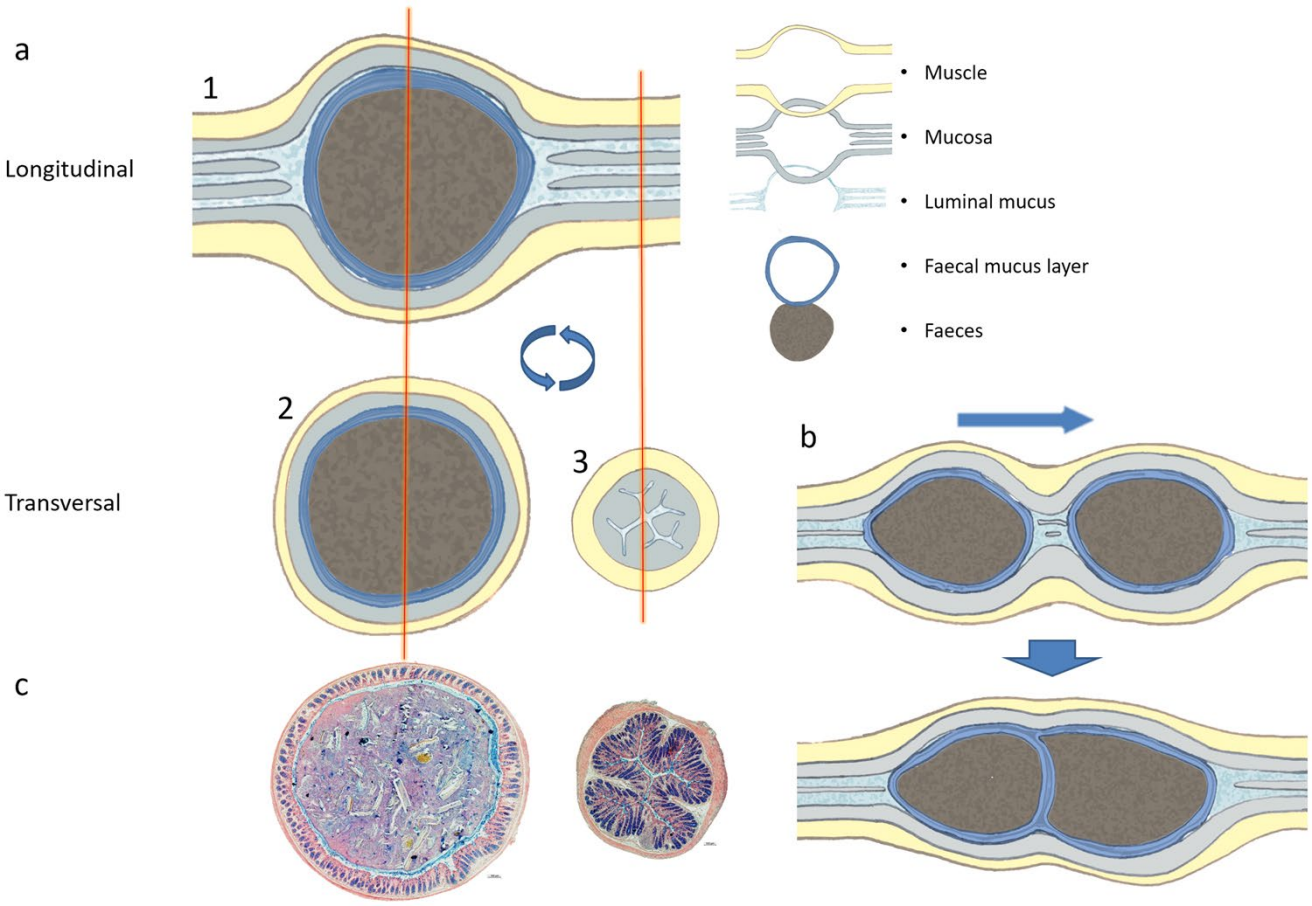
Schematic overview of mucus organisation situations as observed in different sections. (**a1**) In longitudinal sections the mucus layer is found attached to the faecal pellet. (**a2**) In transversal sections, we see the firm mucus layer around the faeces in sections containing faeces. (**a3**) This cannot be observed in transversally sectioned empty colon. (**b**) Further evidence of adherence of the mucus layer to the faecal pellet is provided by the internalisation of the sterile mucus layer inside faecal pellets by collisions between pellets; an observation which can not be explained in the classical model of mucus organisation in which the observed mucus layer is attached to the epithelium. Additionally, the observation of the same mucus layer covering expelled faecal pellets (Fig. 6) indicates clearly that this mucus layer covers the faeces. (**c**) AB/H/E stained sections of full and empty mouse distal colon corresponding to the schematic overview of (**a**).

Longitudinal sections of distal colon reveal that the colonic load is a determining factor in the organisation of the colonic mucus. The model of dual mucus layers covering the colonic epithelium is based on observations in transversal sections of colon containing a faecal pellet^6^. We obtained similar observations in transversal sections (Fig. 1). To our knowledge, this organisation has never been observed in histological sections of untouched tissue lacking faecal pellets, reportedly because the faecal pellet is a necessary factor for mucus conservation during the histological procedures^7^. Our results point out that the faecal pellet is indeed prerequisite for observation of the firm mucus layer, but for different reasons. In longitudinal sections, it is noticed that the observed mucus layers cover the faecal surface instead of the epithelium, and so are not attached to the epithelial surface (Fig. 2). Further supporting this implication, the sterile mucus layer produced by the intestinal mucosa is sometimes found inside a faecal pellet, where it can only have ended up after a collision between two faecal pellets (Fig. 5). Taken together, these data suggest that the use of transversal sections to observe mucus layer organisation has led to the misinterpretation that the faecal mucus layer is attached to the epithelium. Instead, the organisation of the mucus layers seems to be determined by faecal pellet transit within the gut. In our experiments, we found that the sterile mucus barrier of the distal colon is not continuous, but depends on the presence of a faecal pellet. Still, the conditions in empty distal colon are strongly devoid of bacteria, because a clear majority of microbes are removed together with the faecal pellet and its mucus. A schematic overview of our proposed mucus layer organisation in distal colon can be found in Fig. 9.

As supported by literature^8^, mucus is excreted from colonic crypts both in the presence and absence of luminal content, indicating a continuous baseline excretion. In *ex vivo* experiments, mucus produced by the tissue forms a firm inner layer of fresh mucus attached to the epithelium and a loose outer layer of older, more disintegrated mucus^6, 9^. In these conditions, the firm adherent mucus layer of rat colon is reported to be 116 µm^9^, or 101 µm in *in vivo* experiments^17^. In *in vivo* measurements in mice, the firm mucus layer thickness is stated to be 49 µm^6^. In histological sections of empty distal colon, these loose and firm layers are not observed. In these conditions, the lumen is collapsed and the tissue neatly folded, leaving insufficient space to harbour an equally thick mucus layer. Indeed, collapsed distal colon of both rat and mouse has epithelia at around 16 µm from each other, leaving just 8 µm on average per epithelial surface for this supposed mucus layer (Fig. 3). The disparity of these data seems linked to the experimental approach; opening the colon and submerging the epithelial surface in buffer solution, such as usually practiced in *ex vivo* and *in vivo* experiments, might increase the thickness of the observed mucus layer. Mucus swells in a wet environment, based on its properties^18, 19^, so it is important to notice that the humidity levels of the colonic contents in distal colon are normally around 55%, a strong deviation from these experimental conditions. Additionally, it is likely that mucus production and release are increased in response to mechanical and chemical perturbations of the tissue induced by the experiments. Unfolding of the colonic tissue, which is a condition shared between these *in vivo* and *ex vivo* experiments and physiological pellet transit, might influence mucus secretion. An increased mucus secretion can also be induced by the high concentration of microbial products in the faeces, known stimuli of mucus excretion such as short chain fatty acids (SCFAs)^20^, lipopolysaccharides (LPS) and peptidoglycans (PGN)^21^. Specifically LPS was recently shown to trigger crypts to release mucus by activating Nlrp6^22^. These last points are particularly interesting because, as we have shown here, the arrival of the faecal pellet to empty distal colon reintroduces a large microbial presence, and thus increases the presence of LPS and other microbial factors; this could represent an important regulatory mechanism by which the mucus barrier is formed around the faecal pellet. We posit that colonic crypts release additional mucus onto the faecal pellet during its passage, covering it in a mucus layer. Mucus is notoriously difficult to fix for paraffin inclusion. Standard formalin fixation causes the mucus to be completely lost^9^. However, fixation with Carnoy’s solution is currently the most suitable option for conservation of the mucus layers for histological purposes^23^, and although shrinkage does still occur^24^, mucus layers are observed sufficiently well. As discussed earlier, the faecal mucus layer is less hydrated than mucus in an aqueous environment; the resulting increase in density could increase the efficiency of fixation and subsequent staining, which means that a possible highly hydrated surface mucus layer could remain undetected due to restraints inherent in histological techniques. However, we still see that the folding of empty distal colon does not leave enough space for double mucus layers of the thickness classically reported. In the proximal colon, in a similar fashion, the complete absence of a separating mucus layer can not be explained by this type of artefact, instead of empty space left behind after shrinkage, which is normally reported^24^, we see close contact between bacteria and epithelia. Matsuo *et al*.^23^ have shown that it is possible to fix mucus layers covering the intestinal epithelium if they are present, even in the absence of intestinal contents, in apparent contradiction to our findings. In their experiments, surgically removed human intestinal tissue is opened longitudinally and the tissue with mucus layers is fixed using Carnoy’s solution and subsequently histologically visualised. According to our understanding, the patients’ preparation for surgery with laxatives removing the intestinal contents, the opening of the intestinal tube, and the inevitable passage of time between resection and fixation might have led to the formation of attached mucus layers. However, their ability to visualise the mucus layers in these conditions using Carnoy’s solution seems to indicate that the lack of these attached layers in our results is due to actual absence rather than technical artefact. Apparently, surface mucus layers can be absent or present, observable or undetectable, depending on the experimental approach and physiological circumstances. These data show the necessity to further characterise both the regulation and the presence of these secretions in variable conditions, to make sure that a presence is not due to induced formation, and that an absence is not due to technical artefact.

We have no reason to doubt the general functions of the formation of a mucus layer in the distal colon in our adjusted model, which are lubrication^1^ and physical separation of bacteria and epithelium^6^. Additionally, this layer, which covers the entire faecal pellet (see also Fig. 6), in effect isolates the bacteria from the intestinal milieu and confines them to the pellet, as we know from previous research that the mucus layer is mostly impenetrable to bacteria^6^. This explains why no typical bacteria-colonised mucus is observed in empty distal colon (Figs 3b,d and 4). This heavily diminished presence of microbes in empty distal colon is of relevance to the host, because, in the absence of digesta, a large microbial population could be deleterious in the distal colon. Our data suggest that the distal colon contains a mostly transient microbiota, confined to the faeces, which means that most of the resident gut microbiota is hosted higher in the gut, in the proximal colon and cecum, as we have indeed observed. This study, like many other studies on mucus organisation, is performed on rodents, which have markedly drier faeces than humans do. However, the faecal mucus layer is also observed on human stool^12, 13^, so it is likely that this organisation of mucus is shared by rodents and humans. The current widely accepted model has been based on largely the same techniques and experimental practices that we have used in our study, which supports the reliability and relevance of these results.

In proximal colon, released mucus is directly mixed into the chyme, and virtually no mucus is found without bacteria, except at the moment of secretion. This is indicated by the abundance of producing goblet cells in the proximal colon, combined with the lack of a significant excreted mucus barrier. Layer formation in the proximal colon is only observed further towards the transition to distal colon, after the chyme has been shaped into a rudimentary pellet under influence of water extraction by normal functioning of the colon (Fig. 7b). It is likely that the sterile mucus barrier needs a firmer pellet to form, in conditions that prevent it from directly mixing into the chyme. It seems that the classical mucus layer organisation depends on the presence of a firm faecal pellet. This indicates that the faecal mucus layer as reported by Shimotoyodome *et al*.^13^, and again shown by us (Fig. 6), is already, and continuously, deposited on the faeces relatively soon after it passes the proximal colon, and not, as might be expected, starting from the rectum. Simultaneously, we see that mucus mixed with bacteria remains in contact with the epithelium in the collapsed lumen of proximal colon (Fig. 8c,d). In contrast, in collapsed distal colon, we find mucus devoid of bacteria instead (Fig. 3). That the proximal and distal colon do not have an equally thick mucus barrier has also been noted by Ermund *et al*.^24^. The use of transversal sections through a formed pellet has likely prevented them from observing the lack of a mucus barrier in the proximal colon further towards the cecum. To sum up, in the early proximal colon, production of mucus does not lead to a sterile layer, instead mixing into the liquid contents, while in the distal colon, mucus excretion leads to a layer covering the drier faecal pellet.

Because there is a difference in organisation of colonic mucus depending on the location and local stage of transit, the functions we can attribute to colonic mucus secretions depend on these same factors. The intestinal mucus has a modulating effect on the microbiota^25^, together with specific compounds excreted into it, such as defensins, c-type lectins, cathelicidins, and IgA; for reviews on this subject, see for example Duerkop *et al*.^5^, and Liévin-Le Moal and Servin^26^. In proximal colon, the bacteria are mostly growing near to the mucus-producing mucosa, whereas further inwards the luminal contents are made up of coarser materials with less bacteria (Fig. 8). The high density of microbes close to the epithelium indicates that these secretions might support the growth of the microbial population. This could increase the efficiency of the microbial digestive functions; if the population can quickly propagate on easily available substrate, the resulting larger population will have a stronger capacity to utilise less available compounds in the faeces. Additionally, the excretion of mucus in proximal colon, combined with the absorption of water by the mucosa, seems to lead to a change in consistency and shape of the colonic contents (Fig. 7). Mucus being released from goblet cells will sequester water from the environment^18, 19, 27^ while forming a gel, reducing the amount of free water. Mucus released into the chyme can be expected to have a shaping role in the consistency of the colonic contents, where it would serve as a matrix for the microbial community. We hypothesize that mucus released into the chyme in the proximal colon serves to support the intestinal microbiota, and through the gel-like properties of the mucus helps in giving a firmer consistency to the contents. We do indeed see that the bulk of many mouse faecal pellets is made up of microbe-colonised mucus (Figs 1c,d, 4 and 5), indistinguishable from the described^6^ loose mucus layer containing bacteria. Regarding resource and energy efficiency, it seems indeed more expedient to cover the faecal pellet in a thick mucus layer than to cover the entire colonic epithelium, to separate these two components. Clearly, a lubricating mucus layer becomes more crucial as the pellet gets drier and more compact as it progresses through the colon, to prevent damaging abrasion of the tissue. In the proximal colon, when the contents are still liquid, a mucus barrier does not form, even though there is significant production. Direct exposure of the colonic epithelium to the microbiota and digesta is generally expected to cause a strong immune reaction and tissue damage, the prevention of which is often cited as an important function of the mucus barrier. The apparent lack of these effects in these physiological conditions in the proximal colon warrants further investigation.

Our MUCIO approach to histology, in combination with multiple orientation sectioning, has allowed for a comprehensive overview of the evolution of the mucus barrier throughout the colon. Until now, histological results are commonly depicted in single-image microscopic views, which can complicate a thorough understanding of the full results, might underrepresent possible variations throughout the section, and can give rise to a selection bias for ‘suitable regions’ on a slide.

In conclusion, we have shown that a mucus layer is attached to and covers faecal pellets in the distal colon, isolating the faecal bacteria from the intestinal lumen. Mucus is mixed into the chyme and faeces in the proximal colon and in collision events between faeces segments in the distal colon. In the proximal colon, in contrast to the distal colon, no firm mucus barrier is formed until the chyme starts to gain a pellet structure. Before the mucus layer is established, bacteria are in contact with the epithelial surface, and in these conditions, we should reconsider the nature of the barrier function in this region of the colon.

## Materials and Methods

### Animals and sample collection

8 adult male Wistar rats (Janvier, Le Genest St Isle, France) were individually housed in polypropylene cages and offered unlimited access to standard rodent food (Mucedola Global Diet 2018, Harlan, Italy) and water. 8 adult male C57BL/6 mice (Janvier, Le Genest St Isle, France) were housed in polypropylene cages in groups of 4 and offered unlimited access to standard rodent food (Mucedola Global Diet 2018, Harlan, Italy) and water. Rats were euthanized by decapitation, after which 3 cm of distal colon containing faeces was removed and stored in Carnoy’s fixative (60% ethanol, 30% chloroform, 10% glacial acetic acid) overnight. Mice were euthanized by cervical dislocation, after which both 1.5 to 2 cm of distal colon and of proximal colon covering regions with and without contents were removed and stored in Carnoy’s fixative overnight. Mouse faecal pellets were collected directly from the anus, and fixated immediately in Carnoy’s fixative overnight. All animal experiments were performed in accordance with EU directive 2010/63/EU and approved by the local Animal Care and Use Committee of Toulouse Midi-Pyrénées (agreement CEEA-86).

### Tissue processing

The mice and rat tissues were automatically processed by a Shandon™ Excelsior™ ES Tissue Processor using the following program: 2 × 60 min anhydrous ethanol, 2 × 60 min butanol, 480 min butanol, 3 × 80 min paraffin at 60 °C. Tissue samples were included in paraffin blocks using a Thermo Scientific™ HistoStar™ Embedding Workstation. Tissues were oriented for longitudinal or transversal sectioning. 6 µm tissue sections, and 4 µm faecal sections were made using a Microm™ HM 340 E microtome and attached to Thermo Scientific Menzel-Gläser Superfrost® Plus slides.

### Histological staining (AB/H/E staining)

Paraffin embedded sections were deparaffinised using American Mastertech Clearify™ and subsequent passage through an increasingly diluted ethanol-water series, starting with anhydrous ethanol. Staining was performed by 5 min in Hematoxylin, 10 min in running water, 30 min in Alcian Blue solution (pH 3.0) followed by 5 min in running water, 3 min in Eosin, 10 min in 95% ethanol, followed by dehydration in an ethanol series of increasing purity, finishing with dry ethanol, ending with 3 baths of American Mastertech Clearify™, followed by mounting with Diamount mountant.

### Fluorescent *in situ* Hybridisation

FISH staining for all bacteria was performed using probes EUB338I (5′ CGTGCCTCCCGTAGGAGT 3′), EUB338II (5′ GCAGCCACCCGTAGGTGT 3′), and EUB338III (5′ GCTGCCACCCGTAGGTGT 3′). After deparaffinising as described for ‘*Histological staining*’ (see above), the slides were incubated with FISH hybridisation solution (0.9 M NaCl, 20 mM Tris/HCl, 0.01% (v/v) SDS, with 5 ng/µL of EUB338(I/II/III) probes at 48 °C overnight, followed by a washing step with the hybridisation solution without probe at 49 °C for 25 min. Slides were rinsed with demineralised water and briefly air-dried, followed by mounting using ProLong Gold® antifade reagent with DAPI (Thermo Fisher Scientific, USA).

### Imaging; Manual Ultra-high resolution Composite Image Overview (MUCIO)

Manual Ultra-high resolution Composite Image Overview (MUCIO) approach: datasets of 50 to 500 overlapping microscope views covering entire slides were generated by manual microscope photography (single photo resolution: 1280 × 1024 pixels) and stitched together using Microsoft Image Composite Editor (MICE), specifying ‘planar motion’ in the program interface, to best fit the movement of the microscope camera relative to the microscopic slide. The generated composite images contain all the information of the data set in a single ultra-high resolution file. No selection for ‘representative regions’ is necessary, only obvious artefacts (e.g. possibly folded double/heavily damaged sections) are evaded. Samples were imaged using a Nikon Eclipse 90i microscope fitted with a DXM 1200 F Digital Camera. Image sets were taken with 100x or 200x magnification, overlapping and covering complete sections. Resulting images were saved uncompressed in TIFF format. To prevent scaling issues, image resolutions were only downscaled after the appropriate scale bar was integrated. Subsequent downscaling is necessary to facilitate interaction with images for further applications, but close-up views in full quality can be extracted from the original composite.

### Data availability

The data generated and analysed during the current study are available from the corresponding author upon reasonable request.
